# Overall Survival of Elderly Patients Having Surgery for Colorectal Cancer Is Comparable to Younger Patients: Results from a South Asian Population

**DOI:** 10.1155/2017/9670512

**Published:** 2017-07-11

**Authors:** Pramodh Chitral Chandrasinghe, Dileepa Senajith Ediriweera, Thaaqib Nazar, Sumudu Kumarage, Janaki Hewavisenthi, Kemal Ismail Deen

**Affiliations:** ^1^Department of Surgery, Faculty of Medicine, University of Kelaniya, Ragama, Sri Lanka; ^2^Centre for Health Informatics, Biostatistics and Epidemiology, Faculty of Medicine, University of Kelaniya, Ragama, Sri Lanka; ^3^Department of Pathology, Faculty of Medicine, University of Kelaniya, Ragama, Sri Lanka

## Abstract

**Introduction:**

There has been a continuous debate on whether elderly patients with colorectal cancer (CRC) fair worse. The aim of this study is to assess the thirty-day mortality (TDM) and overall survival (OS) of elderly patients undergoing surgery for CRC.

**Method:**

OS between two groups (≥70 versus <70 years) having surgery for CRC was analyzed. Demographics, tumour characteristics, and serological markers were considered as independent factors. Multivariable analysis was done using the Cox proportional hazard model. We also compared overall survival in the elderly versus those <60 and <50 years.

**Results:**

477 patients, 160 elderly (55% male; median age 75, range 70–89) and 317 younger patients (49% male; median age 55, range 16 to 69), were studied. Overall survival in CRC patients ≥70 is comparable to <70 (*P* = 0.45) and <60 years (*P* = 0.08). Poor OS was observed in the ≥70 versus <50 years (*P* = 0.03). TDM in the elderly was poor (*P* < 0.05). Postoperative cardiac complication was the only determinant affecting survival in the elderly (*P* = 0.01).

**Conclusion:**

OS in elderly CRC patients having surgery is not worse compared to <70 and <60 years although the TDM was higher. Postoperative cardiac complications significantly affected OS in those ≥70 compared to those <50 years. Chronological age alone should not negatively influence surgical decision-making in the elderly.

## 1. Introduction

Over 60% of colorectal cancer occurs in those over the age of 65 years in the USA and Europe [1]. Some studies have shown that overall survival in the elderly was worse compared with that in the younger patients [2–4]. The reasons for poor outcome in these studies were advanced stage at presentation and suboptimal management in the elderly [5, 6]. The evidence suggests that the tendency to offer surgical resection and adjuvant therapy was less in the elderly compared with that in the younger patients [7, 8]. Altered physiology due to advanced age and deteriorating organ function seemed to influence decision-making in this group [9]. Furthermore, little data are available regarding the surgical treatment of elderly patients with colorectal cancer in middle-income and resource-limited countries. As the life expectancy improves in these countries such as Sri Lanka [10], more evidence is required to understand factors that affect outcome in elderly patients to improve surgical care. Recent health statistics from Sri Lanka indicate that the population is gradually aging [11]. The “aging index” (proportion between over 60 years population and 0–14 years) has increased from 18.8% to 48.9% from 1981 to 2013 [11]. This implies that in the near future, this region will also be left with a significant elderly population and the disease burden affecting them. The aim of this study was to assess the thirty-day mortality and overall survival in elderly patients undergoing surgery for colorectal cancer and the factors which affect it, in a South Asian center.

## 2. Method

Of patients treated at the university surgical unit at the North Colombo Teaching Hospital from 1995 to 2013, those over 70 years were considered elderly. For each elderly patient, the next two consecutive younger (<70 years) patients were selected as controls by systematic sampling. All patients were worked up and operated by the same team. Standard protocol-based follow-up was employed as previously described and data were entered in a prospective database [12]. All rectal surgeries were accompanied by a diverting ileostomy and the anastomotic leakages reported are the clinically evident leaks. Pathological evaluation of specimens was by one team of pathologists. We compared overall survival of the elderly (≥70 years) with that of the younger patients (<70 years). Sex, presenting symptoms, site of tumour, stage, histological characteristics, CEA level, preoperative albumin level, and postoperative complications were compared between the groups. Further survival analysis was carried out to compare the survival of the elderly with much younger age groups to eliminate the bias in categorization of age by having a single cut-off value (e.g., patient with 69 years and 11 months will be compared with a 70-year-old, where an actual survival difference cannot be expected). For this, we compared survival amongst different age categories with 10-year intervals (≥70 versus <60; ≥70 versus <50). This was done to assess whether the survival of the elderly is comparable with the much younger patient cohorts, as this would make the findings clinically more significant. The thirty-day mortality in each age group was also evaluated and compared between them.

Initially, Kaplan-Meier survival estimate curves were used to compare the survival between the age categories. Multivariable analysis was done using the Cox proportional hazard model where a significant difference was observed in Kaplan-Meier survival estimate curves. The significant individual variables, with a *P* value <0.05 that were identified for these age categories, were subjected to further multivariable analysis to evaluate the adjusted effect. The chi-squared test and Wilcoxon rank sum test were used for group comparisons as appropriate. A *P* value of <0.05 was regarded as significant. Ethical committee approval for the study was obtained from the University of Kelaniya Ethics Review Board.

## 3. Results

### 3.1. Comparison of <70 Years versus ≥70 Years

A total of 477 patients were included in the study. There were 160 elderly patients (age ≥70 years) operated over the study period with a median age of 75 years (range 70–89 years). In the elderly group, 55% were males. Young controls (age <70 years) included 317 patients with a median age of 55 years (range 16–69 years) and 49% being male. The most common site of CRC in both groups was the upper rectum (Table 1). While 9.9% (*n* = 16) of the cancers in the elderly were in the right colon, proportion in the younger group was 17.1% (*n* = 54). Although not statistically significant, a higher proportion of elderly patients presented with intestinal obstruction compared to the younger group (19.25% versus 14.82%; *P* = 0.25).

**Figure 1:**
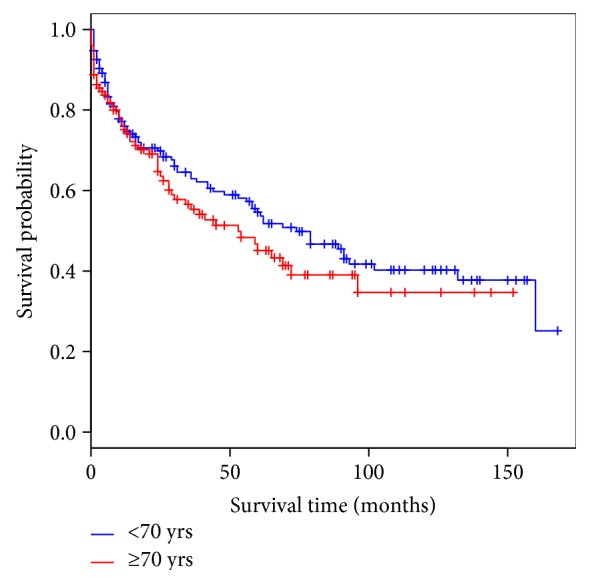
Overall survival in colorectal cancer: <70 years versus ≥70 years.

There was no significant difference in overall survival observed between the elderly patients who underwent surgery for CRC compared with those <70 years old (Figure 1). Except for the preoperative albumin level, other factors were comparable between the two groups (Table 1). A significantly higher proportion of younger patients had a normal preoperative serum albumin level (73% versus 61%, *P* = 0.03). The thirty-day mortality rates in the elderly patients was significantly higher (≥70 years: 12.5% versus <70 years: 6%; *P* = 0.01).

The proportion of advanced cancer (stages III and IV) in both groups was equal (*n* = 58/161 and 123/317; 36% versus 39%, *P* = 0.5). Of the 160 elderly patients, 11.25% (*n* = 18) had metastatic disease at presentation while 7.2% (*n* = 24) of the younger age group was of the same stage. Adjuvant therapy was offered to 19.3% (*n* = 31) of the elderly age group versus 32% (*n* = 101) in <70 years which was statistically significant (*P* = 0.004) [35%; *n* = 74 in <60 years and 35%; *n* = 36 in <50 years age groups].

Postoperative cardiac complications were seen in 7.5% of those over 70 years old compared to those in 1.6% of the younger population (*P* < 0.01). Anastomotic and infective complications did not show a significant difference (Table 1). The 5-year survival rate for the ≥70-year population is 45.1% (95% CI: 35.7–57.0) while for the <70% is 54.6% (95% CI: 47.0–63.4).

### 3.2. Comparison of <60 Years versus ≥70 Years and <50 Years versus ≥70 Years

**Figure 2:**
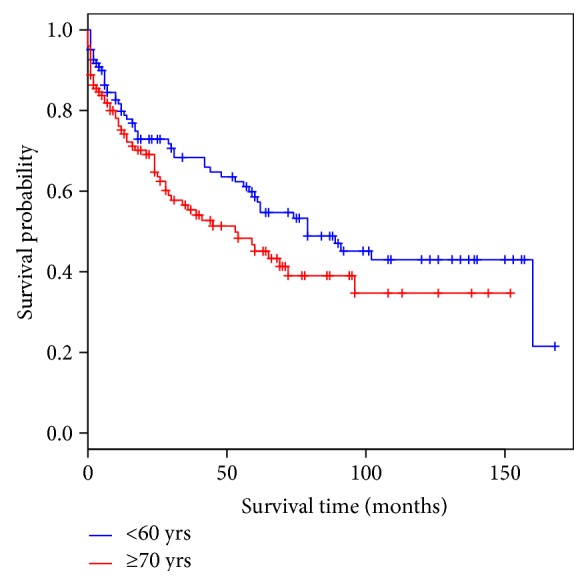
Overall survival in colorectal cancer: <60 years versus ≥70 years.

**Figure 3:**
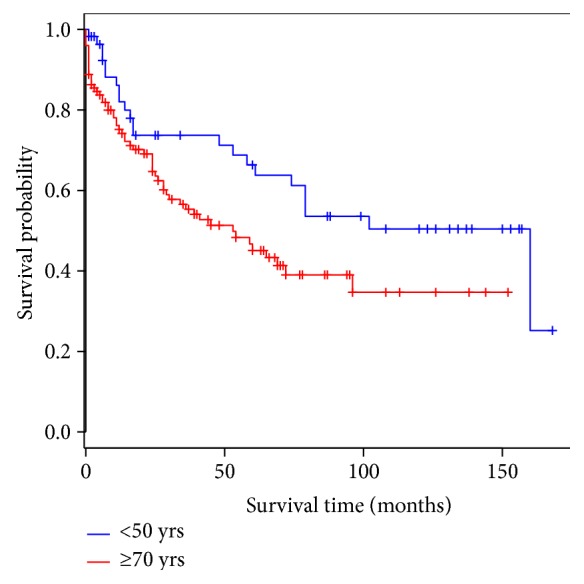
Overall survival in colorectal cancer: <50 years versus ≥70 years.

**Figure 4:**
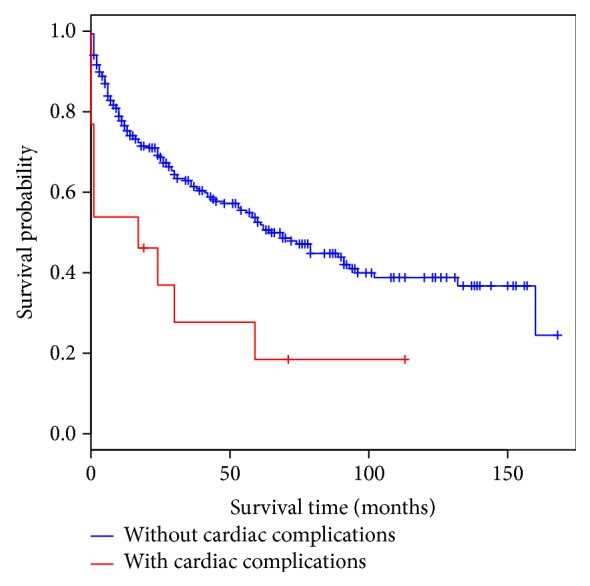
Survival for the total population adjusted for cardiac complications.

In those over 70 years old, overall survival was not significantly different to the survival pattern in those less than 60 years old (Figure 2). The 5-year survival rate for those less than 60 years is 56.8% (95% CI: 49.5–69.4). By contrast, survival in elderly patients is significantly worse compared to those patients less than 50 years of age with colorectal cancer (*P* = 0.03) (Figure 3). The 5-year survival for the <50-year population is 66.3% (95% CI: 54.0–81.4). The thirty-day mortality in the <60-year group was 5.28% (*P* = 0.01) and in the <50-year category was 4.12% (*P* = 0.01) making them significantly lower compared to the elderly. In a multivariable analysis using the Cox proportional hazard method between these two groups, it was shown that irrespective of age the occurrence of a postoperative cardiac complication (*P* = <0.01) was the only determinant, which significantly reduced the probability of survival (Tables 2 and 3). None of the other determinants as considered in Table 1 was significantly different between these two populations. A hazard ratio of 2.8 (95% CI: 1.34–6.0) was observed in the ≥70-year age group with regard to cardiac complications. As the cardiac complication was found to be significant in the elderly patients, a separate analysis was carried out for the whole population with adjustment to age to detect the hazard associated with such a complication. A hazard ration of 2.25 (95% CI: 1.16–4.37) was observed for the whole population when experiencing a cardiac complication irrespective of age category (Figure 4).

## 4. Discussion

Elderly CRC patients have shown an improvement in survival during the past few decades owing to increased detection rates and more aggressive treatment in some settings with advanced surgical care [13]. The upper limit for the definition of elderly has varied from 65 to 80 years in different studies and the cut-off age of 70 years was chosen based on Sri Lankan population demographics [11, 14]. A similar study by Khan et al. analyzing postoperative short-term outcome in a south Asian population has considered 70 years as the cut-off age [15].

The current study showed no difference in survival amongst patients over the age of 70 years compared to those younger than 70. However, significantly lower survival was observed in the elderly when compared with those less than 50 years, and the difference could be attributed to a higher incidence in cardiac complications in the elderly group. Grosso et al. analyzing a European patient population reported that cardiovascular complications in colon cancer patients over 65 years doubled when compared with those in younger patients [16]. Their study also showed a poor survival in the older age group (65 years) with regard to 3- and 5-year survival. In the current study, the age groups less than 60 and 50 years were compared to the elderly as it brings about a considerable age gap between the two groups adding more significance to the age factor as the focus is to evaluate the effect of the chronological age on overall survival.

This study found a 2.8 (95% CI: 1.34–6.0) hazard for those with a cardiac complication in the elderly age group. Thus, survival in the elderly was affected remarkably by such events. While none of 108 patients less than 50 years experienced a cardiac complication, twelve of 161 (7.5%) patients in the over 70-year group had a cardiac-related morbidity. Of the 12 events in the elderly patients, 9 had a fatal outcome. Mitry and colleagues reported in a South Asian population that age is not a significant determinant of short-term complications [12]. Their study observed a significant higher rate of systemic complications in the immediate postoperative period in the elderly although the effect of age was not significant in multivariable analysis. Thirty-day mortality rate in their study was higher in the elderly cohort although they did not perform a multivariable analysis. The current study also confirms the higher rate in short-term mortality in the elderly. These short-term mortality rates are keeping with the observed rates in other similar studies [17, 18]. Authors could not find a previous study from this region comparing long-term survival in elderly patients. The debate continues with regard to the survival of elderly patients after surgery for CRC. In a recent review by Millan et al., the authors discuss the conundrum of surgical decision-making in the elderly patients with CRC [19]. They discuss the concept of using the “frailty index” in decision-making over the chronological age. Also noted in the review is the lower proportion of the elderly receiving adjuvant therapy compared to the younger population. Similar finding can be observed in the current study population as well. Dekker et al. made an interesting observation in a population-based study where the elderly patients who survived the first year after surgery had similar survival as the younger counterparts [20]. Their study concluded that the focus of attention should be to minimize the physiological impact from surgery. High thirty-day mortality rates in the elderly population in the current study with comparable long-term all-cause mortality also indicate similar findings. There is compelling evidence that advance age alone becomes irrespective as a survival determinant in multivariable analysis [21, 22]. There is an increasing trend in taking up aggressive treatment with curative intent in the elderly patients for this reason [23]. In the South Asian population, the initiative to take up aggressive treatment may be still lacking [12]. In the current study, a significant difference is observed in receiving adjuvant therapy between the elderly and the young populations while the tumour stages are comparable. This phenomenon would be due to the conservative attitude taken toward offering this group of patients with aggressive treatment. Lack of supporting evidence with regard to long-term outcome for this population may be contributing to this trend.

## 5. Limitations of the Study

This study does not evaluate disease-free survival amongst the different age categories. Although the intention was to evaluate the all-course mortality, we believe addition of disease-free survival would have been of value. The analysis includes only those who underwent cytoreductive surgery. The data on those who were palliated during the same period is not available and would have added more depth to the analysis in terms of comparing the overall outcome from CRC in different groups of patients. More robust data and in depth analysis of factors ideally with a model setting would provide further evidence in this area.

## 6. Conclusion

This study reveals that there is no difference in overall survival in patients above 70 years compared to those younger. On further analysis, the survival in the elderly group is significantly worse to those younger than 50 years and is influenced by postoperative cardiac complications. The poor thirty-day mortality in the elderly patients may also add up to confirm the effect of cardiac complications in this age group. Therefore, chronological age should not negatively influence decision-making when offering surgical resection in this cohort of patients.

## Figures and Tables

**Table 1:** Factors affecting survival between ≥70 years and <70 years CRC patients.

	Elderly (*n* = 160)	Young (*n* = 317)	*P* value
*Sex*			
Male	88 (55%)	157 (49%)	
Female	72 (45%)	160 (51%)	0.30

*Presentation*			
Intestinal obstruction	31 (19%)	47 (15%)	0.25

*Preoperative serum markers*			
Albumin >3.5 g/L	66 (41%)	166 (52%)	**0.03**
CEA >0.5 ng/L	69 (43%)	118 (37%)	0.10
N/A	25 (16%)	33 (11%)	

*Cancer location*			
Lower rectum	49 (31%)	85 (27%)	0.09
Upper rectum	60 (37%)	130 (41%)	
Left colon	33 (21%)	47 (15%)	
Right colon	16 (10%)	54 (17%)	
N/A	02 (01%)	01 (0.0%)	

*IJCC stage*			
Advanced stage (III/IV)	58 (36%)	123 (39%)	1.00

*Postoperative complications*			
Infective complications	15 (9%)	23 (7%)	0.53
Surgical site infections			
*Superficial*	5	10	
*Deep*	2	4	
*Deep organ space*	0	2	
Other organ systems	8	7	
Cardiac complications	12 (7.5%)	5 (1.6%)	**<0.01**
*Myocardial infarction*	9	2	
*Heart failure*	1	3	
*Arrhythmia*	1	0	
*Endocarditis*	1	0	
Anastomotic complications	3 (2%)	14 (4%)	0.24

**Table 2:** Individual variable analysis survival determinants in ≥70 years versus <50 years CRC patients.

	Estimated coefficient	Standard error	*Z*	*P* value
Age	2.706	0.48857	2.037	0.04
Sex	0.841	0.39359	−0.440	0.66
Bleeding	1.329	0.63587	0.447	0.65
Intestinal obstruction	0.832	0.69147	−0.266	0.79
Albumin	1.055	0.31645	0.168	0.87
CEA	1.004	0.00207	1.726	0.08
Cancer location	0.536	0.32148	−1.940	0.05
Differentiation	0.600	0.44976	−1.135	0.26
Cardiac complications	3.279	0.59450	1.998	0.04
Infective complications	1.156	0.67440	0.215	0.83
Anastomotic complication	0.274	1.06579	−1.216	0.22


**Table 3:** Multivariable analysis on significant variables in ≥70 years versus <50 years CRC patients.

	Estimated coefficient	Standard error	*Z*	*P* value
*Cox proportional method with significant variables (P* < 0.1*;*Table 2)				
Age	1.572	0.368	1.227	0.22
CEA	1.002	0.001	1.794	0.07
Cancer location	0.734	0.197	−1.571	0.11
Cardiac complications	3.399	0.489	2.501	**0.01**
*Final Cox proportional hazard model*				
Cardiac complications	3.3	0.456	2.62	**<0.01**

## Abbreviations

CRC: Colorectal cancer
CEA: Carcinoembryonic antigen.

## Data Access

Complete data set is available for inspection on request by the editor, reviewers, or anyone interested. Data is not shared on a public repository, as the ethical clearance for the matter is not obtained.
